# Functional and Phenotypic Diversity of Microglia: Implication for Microglia-Based Therapies for Alzheimer’s Disease

**DOI:** 10.3389/fnagi.2022.896852

**Published:** 2022-05-26

**Authors:** Yi-Jun Xu, Ngan Pan Bennett Au, Chi Him Eddie Ma

**Affiliations:** ^1^Department of Neuroscience, City University of Hong Kong, Kowloon, Hong Kong SAR, China; ^2^City University of Hong Kong Shenzhen Research Institute, Shenzhen, China

**Keywords:** Alzheimer’s disease, beta-amyloid, neurofibrillary tangles, neuroinflammation, disease-associated microglia (DAM), bipolar/rod-shaped microglia, single-cell RNA-seq (scRNA-seq)

## Abstract

Alzheimer’s disease (AD) is a progressive neurodegenerative disease and is closely associated with the accumulation of β-amyloid (Aβ) and neurofibrillary tangles (NFTs). Apart from Aβ and NFT pathologies, AD patients also exhibit a widespread microglial activation in various brain regions with elevated production of pro-inflammatory cytokines, a phenomenon known as neuroinflammation. In healthy central nervous system, microglia adopt ramified, “surveying” phenotype with compact cell bodies and elongated processes. In AD, the presence of pathogenic proteins such as extracellular Aβ plaques and hyperphosphorylated tau, induce the transformation of ramified microglia into amoeboid microglia. Ameboid microglia are highly phagocytic immune cells and actively secrete a cascade of pro-inflammatory cytokines and chemokines. However, the phagocytic ability of microglia gradually declines with age, and thus the clearance of pathogenic proteins becomes highly ineffective, leading to the accumulation of Aβ plaques and hyperphosphorylated tau in the aging brain. The accumulation of pathogenic proteins further augments the neuroinflammatory responses and sustains the activation of microglia. The excessive production of pro-inflammatory cytokines induces a massive loss of functional synapses and neurons, further worsening the disease condition of AD. More recently, the identification of a subset of microglia by transcriptomic studies, namely disease-associated microglia (DAM), the progressive transition from homeostatic microglia to DAM is TREM2-dependent and the homeostatic microglia gradually acquire the state of DAM during the disease progression of AD. Recent in-depth transcriptomic analysis identifies ApoE and Trem2 from microglia as the major risk factors for AD pathogenesis. In this review, we summarize current understandings of the functional roles of age-dependent microglial activation and neuroinflammation in the pathogenesis of AD. To this end, the exponential growth in transcriptomic data provides a solid foundation for *in silico* drug screening and gains further insight into the development of microglia-based therapeutic interventions for AD.

## Introduction

Alzheimer’s Disease (AD) is the common cause of dementia, which accounts for an estimated 60–70% of all dementia cases throughout the globe (Leng and Edison, 2021). In the United States, an estimated 11.3% of people aged 65 or older are affected by AD-associated dementia. The relative prevalence of AD increased from age 65–74 (5.3%) to ≥85 years (34.6%) (Alzheimer’s Association, 2019). There are approximately 55 million people living with AD or other forms of dementia worldwide, which is expected to increase from 78 million in 2030 to nearly 139 million by 2050 (World Health Organization, 2020). Currently, five pharmacologic treatments for AD are available to improve cognitive function in patients although the improvement is not maintained in the long term (Reisberg et al., 2003; Loy and Schneider, 2006; Cacabelos, 2007; Howard et al., 2012; Birks et al., 2015). However, none of the drugs has been shown to be effectively delayed disease progression and prevented further neuronal damage in the brain. The socioeconomic impact of AD is enormous and the cost of healthcare is estimated to be US$ 1.3 trillion. In the absence of preventive measures and effective treatments for AD, medical cost is believed to exceed US$ 2.8 trillion by 2030 (World Health Organization, 2020). Therefore, there is an urgent need to develop novel therapeutic interventions that can halt the disease progression, especially at the early stage of AD.

It is generally accepted that the deposition of β-amyloid (Aβ) plaques and neurofibrillary tangles (NFTs) caused by Aβ aggregation and hyperphosphorylated tau protein, respectively, are the primary events that lead to progressive loss of cognitive function in AD patients (Brunden et al., 2009; Busche and Hyman, 2020; Walsh and Selkoe, 2020). However, there is compelling clinical evidence suggesting that elevated inflammatory responses might appear much earlier than the deposition of Aβ plaques and abnormal NFTs, which play vital roles in the pathogenesis of AD (Engelhart et al., 2004; Ray et al., 2007; Schuitemaker et al., 2009). For instance, elevated levels of inflammatory proteins, α1-antichymotrypsin (ACT) and interleukin-6 (IL-6), were found in the blood plasma of AD patients before clinical onset of dementia (Engelhart et al., 2004). In another study, an elevated level of C-reactive protein (CRP), a non-specific marker for inflammation, was found in the cerebrospinal fluid (CSF) and serum from patients with mild cognitive impairment (MCI) (Schuitemaker et al., 2009), which is considered as an early stage of AD and other dementia (Petersen, 2011). Similarly, a total of 18 inflammatory proteins were shown to be dysregulated in the blood plasma of pre-symptomatic AD patients (Ray et al., 2007), suggesting inflammation could be a central etiology for AD.

Growing evidence suggests that Aβ deposition and hyperphosphorylated tau proteins induced activation of NLR family pyrin domain containing 3 (NLRP3) inflammasome leading to the production of pro-inflammatory cytokines interleukin-1β (IL-1β) and IL-18. Aβ-induced activation of NLRP3 inflammasome led to the formation of apoptosis-associated spec-like protein containing a CARD (ASC) proteins, resulting in augmented neuroinflammatory responses (Baroja-Mazo et al., 2014; Franklin et al., 2014) and enhanced tau pathology associated with the progressive loss of cognitive function in AD patients (Ising et al., 2019). Recent advances in RNA-sequencing technologies allow in-depth transcriptomic analysis at single-cell resolution, which identified a subset of microglia, namely disease-associated microglia (DAM) (Keren-Shaul et al., 2017; Krasemann et al., 2017; Mathys et al., 2017). In response to Aβ deposition, the resident microglia gradually lost the homeostatic microglia gene signature, and became highly proliferative (Mathys et al., 2017). The progressive transition from homeostatic microglia to DAM is TREM2-dependent (Keren-Shaul et al., 2017; Krasemann et al., 2017). The presence of DAM also induced reactive astrocytes to adopt a neurotoxic A1 phenotype *via* the activation of classical complement cascades (Chen et al., 2020). In this Review, we aim to summarize the current understanding of the role of age-dependent microglial activation and neuroinflammation in the pathogenesis of AD, and provide new insight into the development of microglia-based therapeutic interventions for AD.

## Microglial Activation and Neuroinflammation in the Aging Brain

**FIGURE 1:**
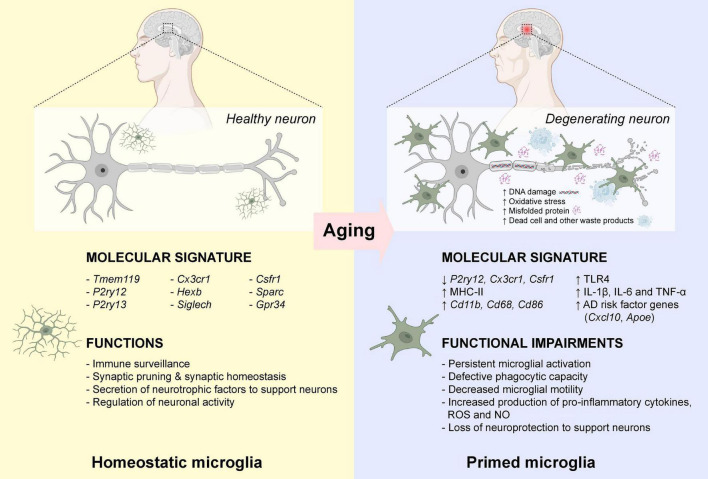
Microglial priming during normal aging. In healthy individuals, microglia are active immune surveillant cells that expressed a wide variety of marker genes for homeostatic microglia. During normal aging, the increased DNA damage and oxidative stress, and the accumulation of misfolded proteins, dead cells and other waste products, induced activation of microglia leading to the down-regulation of homeostatic microglia marker gene expressions. Also, these activated “primed” microglia expressed high levels of major histocompatibility complex II (MHC-II), surface marker proteins (CD11b, CD68, and CD86) and various Toll-like receptors (TLRs). These primed microglia often displayed defective phagocytic capacity and decreased motility toward chemoattractants. The elevated production of neurotoxic pro-inflammatory cytokines lost their neuroprotection to support neurons. Publishing license from BioRender.com.

Neuroinflammation is defined as a cascade of inflammatory responses within the central nervous system (CNS), resulting in sustained production of pro-inflammatory and anti-inflammatory cytokines, chemokines, reactive oxygen species (ROS), and other secondary messengers by resident microglia (McGeer and McGeer, 2010). Microglia are morphologically diverse innate immune cells that are originated from primitive myeloid precursors in the embryonic yolk sac (Ginhoux et al., 2010), which account for approximately 5–12% of the total cell population in the mammalian brain (Dos Santos et al., 2020). As an important player for maintaining homeostasis within the CNS throughout the lifetime, microglia are involved in multiple vital cellular processes including immune surveillance, antigen presentation, debris clearance, neuronal apoptosis, maintenance of synaptic activity, and production of cytokine, chemokine and neurotrophic factors (Tam and Ma, 2014; Heneka et al., 2015; Ransohoff, 2016a; Tam et al., 2016; Au and Ma, 2017, 2022). Impaired microglial function is often associated with various neurodegenerative diseases such as AD (Leng and Edison, 2021). Microglia are highly dynamic immune cells in which they undergo constant changes in morphology in response to the changing microenvironment. Under normal physiological condition, microglia display ramified phenotype with extended branches and processes. Ramified microglia are highly motile which allow them to detect any subtle changes in the microenvironment by extension and retraction of microglial processes (Nimmerjahn et al., 2005). In the presence of endogenous or exogenous insults (mechanical lesion, pathogen, cellular debris, and misfolded protein) that prompt the transformation of ramified “resting” microglia into amoeboid “activated” microglia with thicken and retracted processes, and actively migrate toward the pathological insults (Davalos et al., 2005). Ameboid microglia contribute to the removal and degradation of pathological insults by phagocytosis, endocytosis, and secretion of various inflammatory mediators (Au and Ma, 2017). In general, the inflammatory response is gradually resolved once the pathological insult is removed from the CNS. However, the persistent of DNA damages, oxidative stress, protein misfolding, waste production, and other cellular damages in the CNS microenvironment overtime during aging, microglia became “primed” with exaggerated and heightened microglial responses even toward a minor pathological insult (Norden and Godbout, 2013; Perry and Holmes, 2014; Spittau, 2017). In aged mammalian brains, there is an increased number of amoeboid-like microglia with enlarged cell bodies and shorten processes (Choi et al., 2007; Hwang et al., 2008; VanGuilder et al., 2011). Similarly, microglia also displayed enlarged cell bodies with fewer and shorten processes in aged human brains (Davies et al., 2017), demonstrating that persistent microglial activation occurs during normal aging. Growing evidence suggests that primed microglia induced substantial changes in morphology and inflammatory profiles during the entire course of aging. Aged microglia first showed a significant reduction of homeostatic microglial-specific gene expression such as *P2yr12* (Galatro et al., 2017), *Csf1r*, and *Cx3cr1* (Pan et al., 2020). Moreover, aged microglia showed increased expression of major histocompatibility complex II (MHC II), surface marker proteins (CD11b, CD68, and CD86) and various Toll-like receptors (TLRs) (Perry et al., 1993; Godbout et al., 2005; Griffin et al., 2006; Letiembre et al., 2007; Henry et al., 2009), as well as elevated expression of neuroprotective anti-inflammatory cytokines (IL-4, IL-13, IL-1RA, TGF-β, and IL-10) (Sierra et al., 2007; Luo and Chen, 2012). However, the neuroprotective nature of primed microglia gradually lost over time with increased production of neurotoxic pro-inflammatory cytokines (IL-1β, IL-6, and TNF-α), ROS and nitric oxide (NO) (Li et al., 2018). More importantly, aged primed microglia were more likely to amplify pro-inflammatory responses induced by TNF-α, IL-12, and IL-1β, which further stimulated expansion of primed microglia in the aged brain (Lee et al., 2013). Aged microglia also displayed a defective phagocytotic capacity toward Aβ aggregates (Hickman et al., 2008; Floden and Combs, 2011). The ability of aged microglia to migrate toward an extracellular adenosine triphosphate (ATP), a signaling molecule released by injured neurons, or a laser-induced focal injury was significantly dampened (Damani et al., 2011), possibly due to the dysregulation of actin cytoskeleton dynamics (Galatro et al., 2017). Young microglia returned to the “resting” state within days after focal injury, while aged microglia exhibited persistent microglial activation at the site of injury even weeks after focal injury (Damani et al., 2011). In addition, genes associated with onset of AD such as *Cxcl10* (Bradburn et al., 2018) and *Apoe* (Keren-Shaul et al., 2017) were elevated in the microglia during aging (Pan et al., 2020; Figure 1). To this end, prolonged microglial activation and microglial priming during aging often led to overproduction of neurotoxic pro-inflammatory cytokines results in substantial neuronal damage and cognitive functional impairment in the aged brain, which usually precedes Aβ aggregation and tau pathology (Streit et al., 2009; Navarro et al., 2018).

## Microglial Activation in the Pathogenesis of Alzheimer’s Disease

A long-held hypothesis posits that gradual cognitive function decline in both AD and MCI patients is, at least in part, due to the deposition of Aβ plaques in various brain regions (Rodrigue et al., 2009). Aβ is generated from the amyloid precursor protein (APP) *via* cleavage by β- and γ-secretases (LaFerla et al., 2007). Cleavage and processing of APP proteins are classified into two distinct pathways. Non-amyloidogenic pathways produce beneficial and soluble APP fragment (sAPP-α) *via* cleavage by α-secretase, which involves in the modulation of synaptic plasticity as shown in cultured hippocampal neurons (Rice et al., 2019). Cleavage of APP by α-secretase did not generate Aβ and thus prevented the formation of Aβ plaques (Chen et al., 2017). Amyloidogenic pathways produce APP proteins *via* cleavage by β-secretase (beta-site amyloid precursor protein cleaving enzyme 1; BACE1) and γ-secretase complex which is composed of presenilin-1 (PS-1), presenilin enhancer 2 (PEN2), nicastrin, and APH-1 (anterior pharynx-defective 1) (Kaether et al., 2006; LaFerla et al., 2007). Cleavage of APP by β-secretase and γ-secretase complex generated two main forms of Aβ peptides, Aβ1-40 and Aβ1-42. These Aβ monomer started to aggregate, leading to the formation of neurotoxic soluble Aβ oligomers, and further assembled to form insoluble Aβ fibrils and plaques (Chen et al., 2017). However, the precise mechanisms underlying the formation of Aβ plaques remains unclear. A recent study has demonstrated that increasing cholesterol level in astrocytes facilitated the transfer of cholesterol to neighboring neurons *via* apolipoprotein E (ApoE), resulting in increased Aβ production and plaque formation (Wang et al., 2021). Although deposition of extracellular Aβ plaques is often described as the pathogenic hallmark in AD, emerging evidence suggests that accumulation of intracellular Aβ also exhibited significant neurotoxicity to CNS neurons (D’Andrea et al., 2001; Kienlen-Campard et al., 2002). In various mouse models of AD, intraneuronal accumulation of Aβ often preceded the deposition of extracellular Aβ plaques and subsequent cognitive functional impairment (Oakley et al., 2006; Knobloch et al., 2007; Pensalfini et al., 2014). In the initial stage of AD, Aβ aggregation started to appear within the neuronal cell bodies of large pyramidal neurons and hippocampal neurons (Oakley et al., 2006; Pensalfini et al., 2014). With the increasing loads of intraneuronal Aβ, it leads to the death of the affected neurons and release of Aβ to the extracellular space (LaFerla et al., 2007; Friedrich et al., 2010). These results agreed well with human study that intracellular Aβ accumulation precede Aβ plaque deposition (Gouras et al., 2000). Indeed, a fraction of neurons showed a redistribution of extracellular Aβ plaques into the cell bodies *via* the interaction between Aβ, ApoE and lipoprotein receptor-related protein (LRP) (Bu et al., 2006). The increase in intracellular Aβ loads, particularly the neurotoxic Aβ-42 oligomers within neurons induced defective axonal transport and axonal swelling (Suo et al., 2007), impaired synaptic functions and long-term potentiation (Li et al., 2011), and activation of neuronal apoptosis-related signaling pathways (Ohyagi et al., 2005). These results are consistent with the pathological studies that increased Aβ-42 oligomer is associated with cognitive deficit in AD patients (Lue et al., 1999; McLean et al., 1999).

Another pathogenic hallmark of AD is the aggregation of NFTs which consist mainly of the hyperphosphorylated tau proteins (Brion, 1998; DeTure and Dickson, 2019). Tau is a neuronal-specific microtubule-associated protein which play a pivotal role in maintaining the stability of axonal microtubules, and its activity is tightly orchestrated by tau phosphorylation (Barbier et al., 2019). Since tau-microtubule binding domain is involved in tau-tau interaction, self-aggregation of tau is largely prevented once tau protein is bound tightly with microtubule (Mandelkow and Mandelkow, 2012). However, once tau protein is abnormally hyperphosphorylated under pathogenic conditions and lose its biological activity of microtubule binding. Tau protein detached from microtubule and form aggregates (Mandelkow and Mandelkow, 2012). These abnormally hyperphosphorylated tau proteins gradually accumulated in the cytoplasm of degenerating neurons and formed NFT aggregates in the brain of AD patients (Bancher et al., 1989, 1991). Abnormally hyperphosphorylation of tau protein lost its ability to promote microtubule assembly (Iqbal et al., 1986), and thereby induced the breakdown of axonal microtubules (Lindwall and Cole, 1984; Alonso et al., 1994). The accumulation of NFTs first appeared in entorhinal cortex, which gradually propagated to pyramidal neurons in hippocampal CA1 regions, and finally spread to hippocampus (Braak and Braak, 1991). Interestingly, a recent study confirmed that the pathological tau proteins can be transported from one neuron to another through axonal terminals, suggesting that these dysregulated proteins can be axonally transported *via* active intra-axonal transport throughout the brain (de Calignon et al., 2012). In addition, the extracellular insoluble tau aggregates could induce the accumulation of hyperphosphorylated tau protein in the cytosol of adjacent neurons, leading to the formation of NFTs (Clavaguera et al., 2009). A recent study suggested that the increased accumulation of NFTs in the brain is strongly associated with reduced grey matter volumes and progressive cognitive decline in AD patients (Bejanin et al., 2017).

Increased microglial activation alongside the formation of Aβ plaques and NFTs has been observed in the early stage of AD pathogenesis. Microglia are the first respondent to toxic stimuli that play key roles in maintaining proper synaptic plasticity and circuit integrity (Kettenmann et al., 2011; Wilton et al., 2019). Microglia are traditionally believed to adopt two distinctive microglial phenotypes, namely, the M1 (neurotoxic) and M2 (neuroprotective) subtypes (Heneka et al., 2015; Tang and Le, 2016; Leng and Edison, 2021). M1 microglia, activated by IFN-γ, TNF-α, or lipopolysaccharide (LPS), express pro-inflammatory cytokines such as IL-1β, IL-6 and TNF-α, ROS and nitric oxide (NO). M1 microglia also express NADPH oxidase and high levels of MHC class II that are closely related to increased inflammatory response and neurotoxicity (Cella et al., 1997; Colonna and Butovsky, 2017; Hernández-Espinosa et al., 2019). On the other hands, IL-4 and IL-13 induce a M2 microglial phenotype, which is characterized by the production of anti-inflammatory cytokines (IL-4, IL-10, IL-13, and TGF-β), neurotrophic factors (IGF-1) and increased expression of inflammatory mediators that promote phagocytosis of cellular debris and misfolded proteins, neuronal survival, tissue repair and wound healing processes (Ym1, FIZZ1, and Arg-1) (Cherry et al., 2014; Tang and Le, 2016). At the early stage of AD, activated microglia were able to clear Aβ by phagocytosis (Takata et al., 2010). However, as disease progressed, the increased levels of neurotoxic pro-inflammatory cytokines switched the microglial phenotype from M2 to M1 phenotype with impaired microglial phagocytosis of Aβ plaques (Jimenez et al., 2008). Post-mortem brain tissues in patients at the early stage of AD revealed that an elevated production of pro-inflammatory cytokine IL-1β in activated microglia located in close proximity to senile Aβ plaques (Griffin et al., 1995). An increased level of TNF-α were also detected in the blood sera of AD patients (Fillit et al., 1991). In cultured microglia derived from AD patients, exposure to Aβ induced production of pro-inflammatory cytokines (IL-1β, IL-6, and TNF-α), chemokines (CCL2 and CCL3), and NO (Lue et al., 2001). Therefore M2-to-M1 microglial phenotype switch might contribute to the gradual increase in Aβ plaques and massive neuronal loss at the advanced stages of AD (Tang and Le, 2016). Despite the fact that the elevated production of pro-inflammatory cytokines in microglia is thought to be linked to the exacerbated AD pathologies; however, recent studies demonstrate that overexpression of pro-inflammatory cytokines attenuated the deposition of Aβ. For instance, overexpression of IFN-γ led to a widespread activation of microglia and astrocytes in the hippocampal CA1 and CA3 regions, thereby elevating the production of another pro-inflammatory cytokine TNF-α, and the activation of complement system. The IFN-γ-overexpressing microglia displayed an enhanced phagocytosis which led to a massive reduction in Aβ deposition in a mouse model of AD (Chakrabarty et al., 2010a). Similarly, overexpression of pro-inflammatory cytokines such as IL-1β, TNF-α, and IL-6 induced a widespread activation of microglia and astrocytes with enhanced phagocytic capability, resulting in effective clearance of Aβ plaques in the hippocampus of AD mice (Chakrabarty et al., 2010b,^2011^; Matousek et al., 2012; Rivera-Escalera et al., 2014, 2019). On the other hand, overexpression of anti-inflammatory cytokines IL-4 and IL-10 significantly impaired the phagocytosis of soluble Aβ oligomers by microglia, and increased Aβ deposition in the hippocampus leading to the impairment of cognitive functions in AD mice (Chakrabarty et al., 2012, 2015). Further investigations are required to carefully examine the role of these inflammatory cytokines in the pathogenesis of AD and therapeutic potential for AD.

**FIGURE 2:**
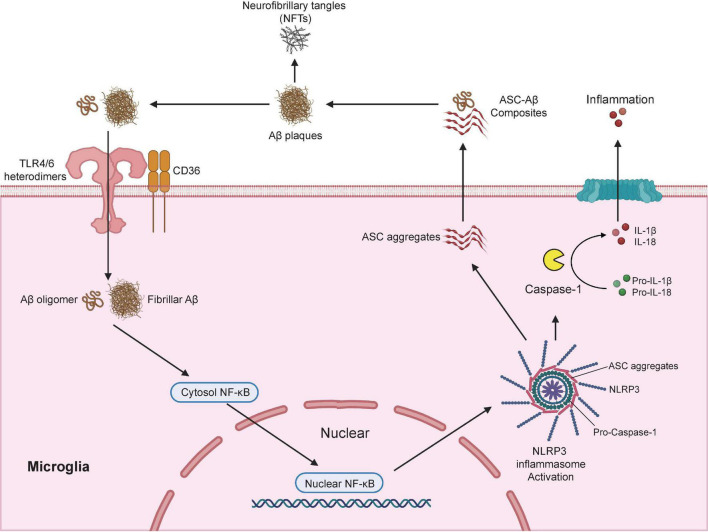
Microglia modulates Aβ plaques and neurofibrillary tangles (NFTs) formation *via* the activation of NLRP3 inflammasome. NLRP3 inflammasome is a multimeric protein complex that comprises a sensor protein NLRP3, an adaptor protein apoptosis-associated spec-like protein containing a CARD (ASC), and an effector protein caspase-1. In AD conditions, microglial surface receptors CD36 cooperates with TLR4-TLR6 heterodimers to recognize and internalize the Aβ oligomers and fibrils, which induces translocation of nuclear factor κB (NF-κB) from the cytosol to the nucleus, and the subsequent activation of NLRP3 inflammasome. Activation of NLRP3 inflammasome leads to the formation of the adaptor proteins ASC aggregates, which bridge the sensor protein NLRP3 and activated the effector protein from pro-caspase-1 to active caspase-1. Activated caspase-1 cleavage of pro-IL-1β and pro-IL-18 into active pro-inflammatory cytokines IL-1β and IL-18. In addition, the speck-like aggregates of ASC proteins could be released to extracellular space and bound to the surrounding soluble Aβ led to the formation of ASC-Aβ composites. The formation of ASC-Aβ composites promoted the Aβ aggregation and served as a major driver for the formation of the neurofibrillary tangles (NFTs). Publishing license from BioRender.com.

Several cell surface receptors in microglia including TLRs, scavenger receptor SCARA1, CD14, CD36, CD47, and α6β1 integrin (Coraci et al., 2002; Reed-Geaghan et al., 2010; Wu and Reddy, 2012; Frenkel et al., 2013) are known to bind soluble Aβ oligomers or Aβ fibrils, which then triggered the nuclear translocation of nuclear factor κB (NF-κB) and the subsequent activation of cAMP response element-binding protein (CREB) (Cunningham, 2013). CREB in turn activated the transcription of various pro-inflammatory cytokines, inducible nitric oxide synthase (iNOS) and cyclooxygenase-2 (COX-2) (Heneka et al., 2015). Recent studies have demonstrated that the activation of NLR family pyrin domain containing 3 (NLRP3) inflammasome is a fundamental event for Aβ-induced inflammatory responses (Heneka et al., 2013; Sheedy et al., 2013). The NLRP3 inflammasome is a multimeric protein complex that contains a sensor protein NLRP3, an adaptor protein PYCARD (also known as apoptosis-associated spec-like protein containing a CARD; ASC) and an effector protein caspase-1 (Swanson et al., 2019). NLRP3 protein consists of three major domains: a carboxyl-terminal leucine-rich repeat (LRR) domain, a central NACHT domain, and an amino-terminal pyrin (PYR) domain (Van Zeller et al., 2021). CD36 acts together with TLR4-TLR6 heterodimers to recognize both soluble and fibrillar Aβ (Stewart et al., 2010; Sheedy et al., 2013), which led to the internalization of Aβ and activation of NLRP3 inflammasome. Activation of NLRP3 inflammasome triggers the cleavage of pro-caspase-1 to active caspase-1, which in turn promotes the cleavage of pro- IL-1β and pro-IL-18 to active pro-inflammatory cytokines IL-1β and IL-18 (Gold and El Khoury, 2015; Figure 2). These studies are consistence with report of substantially increased amount of cleaved caspase-1 in brains from AD patients (Heneka et al., 2013).

Interestingly, genetic ablation of NLRP3 or Caspase-1 significantly reduced Aβ deposition in APP/PS-1 mouse model of AD which was largely due to the enhanced phagocytic capacity in NLRP3-/Caspase-1-deficient microglia to actively engulfing Aβ plaques. NLRP3/Caspase-1-deficient microglia also displayed elevated productions of anti-inflammatory cytokines including IL-4, Arg-1, and FIZZ-1, and reduced expression of NOS2. As a result, APP/PS-1 transgenic mouse with NLRP3-/Caspase-1-deficiency exhibited a significant improvement in spatial memory function (Heneka et al., 2013). After NLRP3 inflammasome activation, adaptor protein ASC bridges the sensor protein NLRP3 *via* PYD domain, leading to the formation of ASC fibrils (Lu et al., 2014). ASC fibrils then recruit the effector protein caspase-1 *via* its CARD domain for caspase activation, which leads to the formation of speck-like aggregates of ASC proteins (Masumoto et al., 1999). Once the ASC speck-like aggregates are formed, they are released into the extracellular space and taken up by adjacent myeloid cells such as microglia to further propagate and amplify inflammatory responses (Baroja-Mazo et al., 2014; Franklin et al., 2014). ASC speck-like aggregates are found within the activated microglia and the extracellular space in the hippocampus of AD patients and APP/PS-1 mouse model of AD. Exposure to soluble Aβ stimulated the production and release of ASC speck-like aggregates in cultured primary mouse microglia (Venegas et al., 2017). Extracellular ASC speck-like aggregates bind to the surrounding soluble Aβ and promote the formation of Aβ aggregates in APP/PS-1 transgenic mouse. Intrahippocampal injections of recombinant ASC speck-like aggregates into the APP/PS-1 transgenic mouse facilitated the propagation of Aβ deposition. Genetic deletion of ASC or by administration of neutralizing antibodies against ASC proteins significantly reduced the deposition of Aβ and improved cognitive functions in APP/PS-1 transgenic mice (Venegas et al., 2017). A recent study also confirmed that NLRP3 inflammasome contributed to the development of tau pathology (Ising et al., 2019). Activation of NLRP3 inflammasome (as reflected by the increased level of cleaved caspase-1) and ASC speck-like aggregates were found in the hippocampus of patients with frontotemporal dementia (FTD) (Ising et al., 2019) as well as in a mouse model of tau pathology (Tau22 mice which expressed human tau mutation for FTD) (Schindowski et al., 2006). Genetic ablation of NLRP3 markedly inhibited the hyperphosphorylation of tau protein and rescued spatial memory impairment in Tau22 mice. More importantly, intracerebral injection of Aβ-containing brain homogenate into Tau22 mice exaggerated tau hyperphosphorylation in the hippocampus, which was completely prevented when NLRP3 was deleted from Tau22 mice (Ising et al., 2019), suggesting NLRP3 inflammasome is necessary to drive Aβ-induced tau pathology (Figure 2). A recent in-depth transcriptomic analysis confirmed that Aβ deposition and microglial activation concurrently drive pathogenic tau spread in different brain regions in AD patients (Pascoal et al., 2021). Taken together, these studies highlight the potential of pharmacological targeting the activation of NLRP3 inflammasome as an attractive therapeutic strategy for the treatment of AD.

## Disease-Associated Microglia and Its Involvement in the Pathogenesis of Alzheimer’s Disease

**FIGURE 3:**
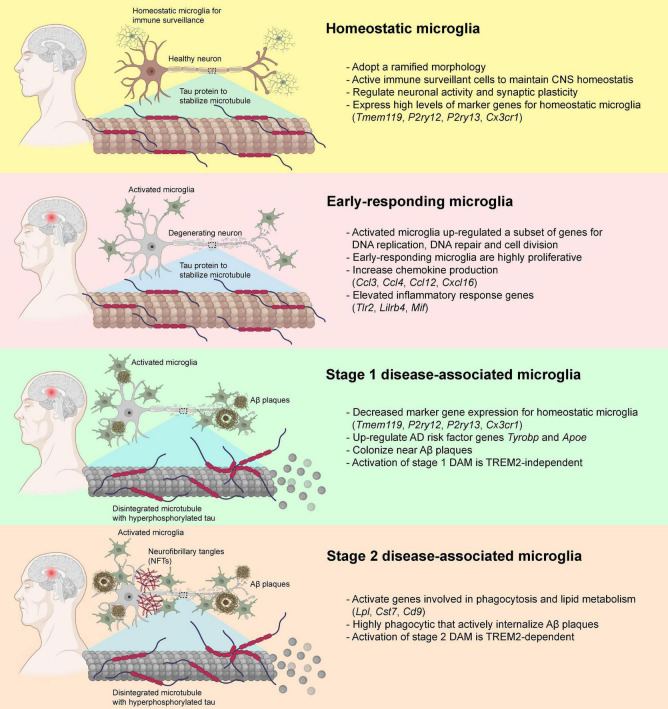
Disease-associated microglia (DAM) and their transformation during the pathogenesis of AD. In healthy individuals, microglia usually adopt a ramified morphology and expressed a diverse variety of marker genes for homeostatic microglia. In the early pathogenesis of AD that preceded the deposition of Aβ plaques and neurofibrillary tangles (NFTs), the homeostatic microglia transformed into a highly proliferative state and elevated the production of various chemokines and other inflammatory genes. When Aβ plaque loads gradually increased, some of the microglia down-regulated the expression of the homeostatic microglial marker genes, transformed into stage 1 DAM with elevated expression of *Tyrobp* and *Apoe via* TREM2-independent pathways, and started colonizing near the Aβ plaques. With the increase in Aβ and NFT deposition, the stage 1 DAM slowly transformed into fully activated stage 2 DAM *via* TREM2-dependent pathways. These stage 2 DAM were highly phagocytic and involved in the active internalization of Aβ plaques *via* activation of genes involved in phagocytosis and lipid metabolisms. Publishing license from BioRender.com.

The classic M1-M2 dichotomy has been used traditionally to describe the microglial activation states when purified microglial cells are exposed to stimuli in cultures. However, M1-M2 phenotypic states might not emerge as isolated pure phenomena in animal studies, suggesting the need to reconsider the rather simplified M1-M2 phenotypic switching concept (Ransohoff, 2016b). In the past decade, transcriptomic analysis at single-cell resolution identified functionally and phenotypic distinct microglial subtypes that closely associate with the pathogenesis of AD, namely the disease-associated microglia (DAM) (Keren-Shaul et al., 2017; Krasemann et al., 2017). Homeostatic microglia gradually acquire the state of DAM during the disease progression of AD (Keren-Shaul et al., 2017; Krasemann et al., 2017; Mathys et al., 2017; Chen et al., 2020). A recent study utilized a well-established mouse model of AD that expressed five human familial AD gene mutations (5xFAD) to examine the temporal transcriptomic changes at single-cell resolution (Keren-Shaul et al., 2017). The Aβ deposition reported in 5xFAD mouse model starting at 1.5 months with profound Aβ deposition in the cortical and hippocampal regions at around 2 months (Oakley et al., 2006; Eimer and Vassar, 2013). Two DAM subtypes with distinct transcriptomic profiles were identified during the disease progression of AD in 5xFAD mice (Keren-Shaul et al., 2017). Before the disease onset, homeostatic microglia expressed high levels of microglia-specific marker genes including *P2ry12*, *Cx3cr1*, and *Tmem119* (Hickman et al., 2013; Bennett et al., 2016), and the expression was gradually declined when microglia were activated during the disease progression (Keren-Shaul et al., 2017). Transition from homeostatic to DAM state seems to be a two-step process (stage 1 and 2) in which homeostatic microglia first transition to an intermediate stage (stage 1 DAM) in a TREM2-independent manner, followed by a TREM2-dependent transition to stage 2 DAM (Figure 3). The upregulated genes in stage 1 DAM, including AD-associated genes such as *Tyrobp* and *Apoe*, which occurs during the early stages of disease. Stage 2 DAM usually appear in the advanced stage of AD characterized by the upregulation of *Cst7*, *Lpl*, and *Trem2*. These DAMs not only increase gene expression responsible for inflammatory responses, but also genes for phagocytosis and endocytosis. In both 5xFAD mice and post-mortem human AD brains, a significant subpopulation of DAM was localized in close proximity to the Aβ aggregates which enabled active internalization of Aβ aggregates (Keren-Shaul et al., 2017). By single-nucleus RNA (snRNA) sequencing, a comprehensive transcriptional analysis on *Trem2*-deficient 5xFAD mice and AD patients who carried the *TREM2* variants was performed. The presence of TREM2-dependent DAM was confirmed in patients with AD carrying the *TREM2* variant. Genetic deletion of *Trem2* markedly reduced the microglial density in 5xFAD mice, and reduced the expression of DAM signature genes, suggesting that Aβ-pathology-induced microglial expansion is partially TREM2-dependent (Zhou et al., 2020). However, the molecular mechanisms underlying the transition from homeostatic microglia to DAMs remains elusive that requires further investigation.

Similarly, two distinct phenotypes (early and late phases) of microglia were identified during the disease progression of AD using an inducible transgenic mouse line of p25 (Mathys et al., 2017). At the early stage of AD (i.e., 1 week after p25 induction) when intraneuronal Aβ deposition was absent (Cruz et al., 2006; Sundaram et al., 2012), the early-responding microglia upregulated a subset of genes involved in the cell cycle, DNA replication and cell division, which switched the cells into a highly proliferative state and began to colonize the hippocampal CA3 and dentate gyrus subregions (Mathys et al., 2017). After initial stage of microglial proliferation in response to neurodegeneration events, microglia switch from the early-responding phenotype to a late-responding phenotype with upregulation of immune response-related genes (i.e., 2–6 weeks after p25 induction) (Mathys et al., 2017) at the time when intraneuronal Aβ level was markedly increased accompanied by widespread neuronal and synaptic loss in the hippocampus (Cruz et al., 2006). These late-responding microglia expressed subsets of genes encoded for MHC class I and II components, interferon-responding genes, and various inflammatory cytokines and chemokines (Mathys et al., 2017). Also, they elevated the expression of many marker genes for both stage 1 and 2 DAMs, suggesting a substantial overlap of gene expression profiles between DAMs and late-responding microglia. Interestingly, most genes that expressed exclusively in early-responding microglia showed little overlap with those genes expressed in stage 1 and 2 DAMs, indicating that the early-responding microglia appear to precede the activation of DAMs (Keren-Shaul et al., 2017; Mathys et al., 2017; Figure 3).

A subsequent study further confirmed that the activation of TREM2-APOE signaling pathways in DAM is essential for the switch of homeostatic microglia to DAMs during the pathogenesis of various neurodegenerative diseases including AD, amyotrophic lateral sclerosis (ALS) and multiple sclerosis (MS) (Krasemann et al., 2017). The apoptotic neurons act as the driving force for the activation of DAM. The homeostatic microglia rapidly migrated toward the apoptotic neurons, transformed into amoeboid-like DAMs and to actively engulfing the apoptotic neurons with elevated expression of Apoe. The upregulation of Apoe in DAMs suppresses the major transcription factors of homeostatic microglia (*Mef2a*, *Mafb*, and *Smad3*) and TGF-β-mediated anti-inflammation. Genetic ablation of *Apoe* or its upstream regulator *Trem2* restored homeostatic microglia gene signature (i.e., *P2ry12*, *Gpr34*, *Tmem119*, *Tgfbr1*, and *Csf1r*), while the genes essential for the DAM phenotypic transformation were suppressed (*Trem2*, *Axl*, *Clec7a*, *Csf1*, *Itgax*, *Cd34*, and *Apoe*) (Krasemann et al., 2017). *Apoe*-deleted homeostatic microglia displayed an effective phagocytic response to engulf and degrade Aβ plaques in a mouse model of AD coexpressing mutant amyloid precursor protein (APP) and presenilin 1 (PS-1) (Krasemann et al., 2017). Interestingly, the elevated expression of *Apoe* was negatively correlated with the loss of homeostatic microglia gene signature as shown in various mouse models of neurodegenerative diseases (two AD mouse models and one ALS mouse model), and in post-mortem brains from AD patients (Sobue et al., 2021). An exhaustive gene expression profiling study on a widely used mouse model of AD that expressed mouse APP with three AD-associated mutations (Swedish mutations in exon 16, and Beyreuther/Iberian and Arctic mutations in exon 17) (Saito et al., 2014), showing the heterogeneity in both homeostatic and reactive microglia. The authors uncovered a major population of reactive microglia, namely activated response microglia (ARMs), which displayed multifunctional gene response and is part of the normal evolution of microglia during the aging process. The presence of amyloid plaques in APP mice accelerated the switching of homeostatic microglia to ARMs. One of the known AD risk genes such as *Apoe*, were highly upregulated in ARMs cells. Genetic deletion of ApoE significantly reduced the number of microglia expressing ARMs signature genes, suggesting that the strong association of AD genetic risk factors, and the activation and function of ARMs (Sala Frigerio et al., 2019). It suggests that ApoE dysregulation plays a key role in the transition of homeostatic microglia to DAM in neurodegenerative diseases.

More recently, it has been suggested that a specific subtype of microglia termed “human Alzheimer’s microglia (HAM)” was identified in post-mortem human brain and highly relevant for the pathogenesis of AD (Srinivasan et al., 2020). In total, 66 genes (45 up-regulated and 21 down-regulated) were differentially expressed in microglia within the superior frontal gyrus (a region for visuospatial cognitive functional impairment) of AD patients. Interestingly, a large number of genes that were upregulated in HAM overlapped significantly with genes upregulated in aged human cortical microglia (Galatro et al., 2017), suggesting similarities between AD and age-related neuropathology. Several upregulated genes in the HAM including APOE, LSR (lipolysis-stimulated lipoprotein receptor) and ARSA (lysosomal enzyme), which are associated with lipid and lysosomal signaling pathways (Srinivasan et al., 2020). In addition, two distinct microglia subclusters were identified in post-mortem human brains that were strongly associated with either Aβ plaques (i.e., AD1-microglia) or hyperphosphorylated tau (i.e., AD2-microglia) by snRNA sequencing. In AD1-microglia, APOE and TREM2 were significantly up-regulated. Notably, of the 63 AD genetic risk factors, 15 were highly upregulated (i.e., TREM2 and APOE) in AD1-microglia, while only six genes were found to have moderate changes in gene expression in AD2-microglia, suggesting the genetic risk of AD is primarily associated with Aβ plaques pathology (AD1-microglia), but not with phospho-tau pathology (AD2-microglia) (Gerrits et al., 2021). Increasing evidence suggests that APOE is the major genetic risk factor of late onset and sporadic AD, which accounts for 95% of total AD cases (Rebeck et al., 1993; Saunders et al., 1993; Coon et al., 2007). In mice, a number of DAM signature genes including *Apoe*, *Ch25h*, *Lpl*, *Ctsb*, and *Atp6v0d2* were highly upregulated and involved in the lipid metabolism (Keren-Shaul et al., 2017). Taken together, transcriptomic studies in both AD patients and mouse models of AD highlight the pivotal role of microglia in lipid metabolism during disease progression (Matsuzaki et al., 2011; Kang and Rivest, 2012). Therapeutic interventions targeting TREM2-APOE-mediated lipid metabolism could switch the HAM or DAMs to homeostatic microglia and to halt AD progression (Nugent et al., 2020; Fitz et al., 2021).

Apart from DAM, a spatially resolved transcriptomics study reveals that A1 astrocytes are the major driver for AD progression in response to Aβ deposition (Chen et al., 2020). In APP mice, which exhibited substantial activation of microglia and astrocytes, as well as Aβ deposition in both cortical and hippocampal areas (Saito et al., 2014). A subset of plaque-induced genes (PIGs) was gradually increased in both DAMs (*Apoe*, *Trem2*, *Tyrobp*, *Cstd*, and *Lyz2*) and A1 astrocytes (*Gfap*, *B2m*, and *C4b*) accompanied by an increase in Aβ accumulation across different brain regions (Chen et al., 2020). Temporally, increased Aβ deposition in the early onset of AD first triggered the activation of microglia which expressed high levels of DAM signature genes (i.e., C1qa and C1qb) (Chen et al., 2020). The elevated expression of C1qa and C1qb, and the activation of classical complement system in DAMs surrounding the Aβ plaques induced astrocytes to adopt neurotoxic A1 phenotype (Liddelow et al., 2017) by increasing A1 astrocytes signature gene expression (*B2m* and *Gfap*) (Chen et al., 2020). A subsequent co-expression network analysis of PIGs revealed that DAM signature genes (*Trem2* and *Tyrobp*) had the strongest connection to the A1 astrocytes signature genes (*Cstd*, *B2m*, and *Apoe*) (Chen et al., 2020). These studies further support the notion that intercellular crosstalk between DAM and A1 astrocytes is crucial for AD progression (Liddelow et al., 2017). A1 astrocytes have been shown to be highly neurotoxic that led to a substantial neuronal and synaptic loss in cultured CNS neurons (Carpanini et al., 2019). Pharmaceutical blockade of the classical complement cascade activated in DAM might prevent the A1 astrocyte phenotypic switch that could foster neuroprotection (Carpanini et al., 2019). A1 astrocytes were found in the post-mortem prefrontal cortex of AD patients (Liddelow et al., 2017). In another study, a subset of reactive astrocytes, namely disease-associated astrocytes (DAAs), is identified in which the expression profile of DAA is largely overlapped with A1 astrocytes. The abundance of DAAs substantially expanded (up to 40% among the total astrocyte population) during the AD progression in 5xFAD mice (Habib et al., 2020). Interestingly, the population of DAA also underwent gradual expansion in aged mice, as well as in post-mortem brains from aged human (Habib et al., 2020). This is consistent with other study that astrocytes gradually adopted an A1-like astrocytic phenotype during normal aging (Clarke et al., 2018).

Recent advances in transcriptomic analysis on microglial populations from AD patients and various AD mouse models have revolutionized our understanding on the roles of microglia in the pathogenesis of AD. A general observation from the above transcriptomic studies is that resident microglia gradually lost their gene signatures during aging and AD progression. Although the functions of these microglia signature genes are yet to be determined, it is believed that they are involved in the CNS-specialized functions which separate microglia from other blood-borne myeloid cells. Loss of the microglia gene signature might affect cellular homeostasis in the CNS during normal aging and AD progression (Friedman et al., 2018). TREM2-mediated signaling pathways regulate transition from homeostatic microglia to DAM (Keren-Shaul et al., 2017). Although major controversy regarding the TREM2-dependent DAM activation in fostering neuroprotection in AD still remains. Genetic deletion of TREM2 impaired the transition from intermediate state DAM to DAM with enhanced phagocytic capacity (Keren-Shaul et al., 2017). Further study revealed that AD Mice with TREM2 haplodeficiency or TREM2-deletion led to the formation of more diffused Aβ plaques, which increased the area of contact between Aβ plaques and adjacent neurons resulting in exaggerated axonal damages in the surrounding tissues (Yuan et al., 2016b; Meilandt et al., 2020). In contrast, TREM2 is required for the compaction of Aβ into dense plaques by microglia that limits Aβ plaque diffusion and exposure to minimize amyloid-related neuronal damage (Yuan et al., 2016b). In 5xFAD mice, overexpression of TREM2 markedly promoted the phagocytic capacity of resident microglia and reduced Aβ deposition. TREM2 overexpression inhibited the activation of classical complement cascade significantly in 5xFAD mice as well as attenuating A1 astrocyte-induced inflammatory responses. In addition, TREM2 overexpression upregulated genes that involved in microglial phagocytosis (*Lgals3*), anti-inflammatory responses (*Postn*), and microglial survival (*Spp1*) in 5xFAD mice (Lee et al., 2018). The production of neuroprotective anti-inflammatory cytokines (arginase-1, IL-10, and Ym1) was elevated and the production of neurotoxic pro-inflammatory cytokines (TNF-α, IL-6, and IL-1β) was reduced by TREM2 overexpression. In a recent study, it has been shown that TREM2 could be a valuable therapeutic target for the treatment of AD since TREM2 overexpression rescued cognitive impairments in APP/PS1 mice by inhibiting microglia-mediated neuroinflammation (Ruganzu et al., 2021).

Interestingly, there was very little overlap between DAM gene signatures identified from different mouse models of AD and HAM gene signatures from human AD patients (Srinivasan et al., 2020). One possible explanation is that most of the AD mouse models overexpress one or multiple gene mutations for familial AD, which accounts for only 5% of AD patients worldwide (Piaceri et al., 2013). The microglial responses to familial AD mutations might be different in sporadic AD. Another possible explanation could be that the mechanisms underlying inflammatory responses to microglial activation in mouse models of AD and AD patients are very different indeed. We should note that AD is clinically heterogeneous in disease progression that might also account for the discrepancy between DAM and HAM gene signatures across studies (Leng and Edison, 2021). For instance, a meta-analysis comparing microglial expression profiles from various animal models of AD, including mouse models that overexpressed familial AD mutations (5xFAD and APP/PS1 mice) and mouse models of tau pathology, revealed elevated expression of core neurodegeneration-related genes across the various mouse models. The core of neurodegeneration-related genes included DAM signature genes such as *Apoe*, *Itgax*, *Hif1a*, *Ctsb*, *Ctsl*, and *Ctsz*, as well as genes that regulated lysosomal activities (Friedman et al., 2018). Nevertheless, these transcriptomic studies all propose the key roles of ApoE in the pathogenesis of AD, which required further investigation regarding its functions in regulating the microglial activation state. Interestingly, a recent study demonstrated that genetic ablation of ApoE in microglia did not affect the microglial transcriptional shift to a DAM state; however, deletion of ApoE in microglia slightly increased Aβ deposition and reduced the number of pre- and post-synaptic vesicles in hippocampus of 5xFAD mice (Henningfield et al., 2022). It therefore suggests that microglial-specific ApoE expression could be necessary for effective clearance of Aβ plaques and maintenance of functional synapses in AD.

## TREM2-DAP12 Signaling Pathway in Alzheimer’s Disease

TREM2 is an immunoglobin superfamily receptor present on myeloid cells such as dendritic cells, osteoclasts, macrophages and microglia (Wang et al., 2015a), and signal through DAP12 (also known as TYROBP). DAP12 is one of the immunoreceptor tyrosine-based activation motif (ITAM)-bearing polypeptides. ITAMs were first identified in the immune system, where they play key roles in cellular response including cell adhesion, migration, differentiation proliferation and phagocytosis (Underhill and Goodridge, 2007). Once DAP12 binds to TREM2, the non-receptor tyrosine kinase named Src phosphorylates the ITAM of DAP12, leading to recruitment and activation of Src homology 2 (SH2) domains of another protein tyrosine kinase named spleen tyrosine kinase (SYK) (Konishi and Kiyama, 2018), which activates a wide range of downstream signals and key cellular responses such as microglial phagocytosis (Linnartz et al., 2010). The ITAM-mediated activation of microglial phagocytic processes is counter-regulated by immunoreceptor tyrosine-based inhibition motif (ITIM) signaling molecules such as sialic acid-binding immunoglobulin superfamily lectins (Siglecs) (Linnartz-Gerlach et al., 2014). Several ITAM and ITIM-containing proteins are known to be involved in the pathogenesis of AD, including DAP12, SYK and CD33 (Ma et al., 2015). TREM2-DAP12 complex played an indispensable role in mediating the microglial phagocytic processes and cytokine production (Konishi and Kiyama, 2018), and the formation of TREM2-DAP12 complex is required for microglial activation to limit the accumulation and diffusion of Aβ plaques (Wang et al., 2015b,^2016^; Yuan et al., 2016a). In mouse models of AD, overexpression of DAP12 in microglia reduced the Aβ deposition and elevated the level of phosphorylated Tau in the cerebral cortices. Interestingly, overexpression of DAP12 induced up-regulation of other DAM genes such as *Apoe*, *Itgax*, *Axl* and *Tgf-*β in the mouse model of AD, suggesting that DAP12 is essential for DAM phenotypic transformation (Audrain et al., 2021). In cultured primary microglia and AD patients, exposure to soluble Aβ peptides/plaques induced the formation of large and persistent stress granules within microglia. SYK proteins were recruited to these stress granules and thereby led to the impairment of microglial phagocytosis and augmented ROS production. Pharmaceutical blockade of SYK protein suppressed the formation of stress granules and attenuated ROS production but failed to restore the microglial phagocytic capacity (Ghosh and Geahlen, 2015). Interestingly, recent studies showed that selective blockade of SYK proteins markedly decreased Aβ deposition and suppressed the hyperphosphorylation of Tau across different mouse models of AD (Paris et al., 2014; Yamaguchi et al., 2020), suggesting that SYK activity might be an attractive therapeutic target for AD.

CD33 is an ITIM-containing protein that is linked to the late-onset AD in human (Griciuc et al., 2013; Wißfeld et al., 2021). A genetic variant of CD33 gene (rs24162737) is associated with the increased risk factor of AD (Lambert et al., 2013). In cultured microglia, overexpression of CD33 largely reduced the uptake of insoluble Aβ, which might positively correlate with the increased Aβ deposition with elevated immunoreactivity of microglial CD33 in AD patients (Griciuc et al., 2013). Genetic deletion of CD33 markedly enhanced the microglial phagocytosis of Aβ aggregates *in vitro* (Griciuc et al., 2013; Wißfeld et al., 2021), and reduced Aβ deposition *in vivo* leading to improved cognitive functions in mouse models of AD (Griciuc et al., 2013, 2019). Clec7a (also known as Dectin-1) is a transmembrane non-TLR pattern-recognition receptor that signal *via* a ITAM-like motif in its cytoplasmic tail and induces microglial activation *via* activation of Src and Syk family kinases (Brown, 2006; Goodridge et al., 2011). In developing mouse brain, a large population of Clec7a-positive microglia was found in corpus callosum and cerebellar white matter. Clec7a-positive microglia were mostly amoeboid in shape and showed a higher phagocytic capacity than the Clec7a-negative microglia (Li et al., 2019). In AD mice, Clec7a was up-regulated in a TREM2- and APOE-dependent manner (Krasemann et al., 2017). The Clec7a-positive microglia were found adjacent to Aβ plaques (Keren-Shaul et al., 2017; Krasemann et al., 2017), suggesting that Clec7a is functioned as a modulator of microglial phagocytosis.

## Bipolar/Rod-Shaped Microglia in Alzheimer’s Disease

Over the past decades, much effort has been attributed to characterize ramified (resting) and amoeboid (activated) microglia in various neurological disorders that might be an oversimplification. Apart from the classical subdivision of microglia into ramified and amoeboid microglia, bipolar/rod-shaped microglia also appeared in the brains under several pathological conditions (Ziebell et al., 2012; Taylor et al., 2014; Au and Ma, 2017, 2022; Bachstetter et al., 2017; Edler et al., 2018; Holloway et al., 2020). In fact, bipolar/rod-shaped microglia was the first form of activated microglia characterized by Nissl in 1899 (Nissl, 1899). This form of microglia were “strung-out,” with extremely slim cell bodies and indefinitely long processes (Nissl, 1899). Subsequent studies also found that bipolar/rod-shaped microglia were present in cerebral cortices of patients with paralytic dementia, typhus or syphilis infections, and sleeping disorders (Nissl, 1899; Spielmeyer, 1923). However, the functional role of bipolar/rod-shaped microglia remains largely unexplored until recently. In an experimental model of traumatic brain injury using a midline fluid percussion injury, ramified microglia quickly transformed into bipolar/rod-shaped microglia and formed end-to-end alignment in close proximity with injured axons at the primary somatosensory barrel field (S1BF) region of rat cerebral cortex at days 1–7 post-injury (Ziebell et al., 2012; Taylor et al., 2014). These trains of bipolar/rod-shaped microglia also displayed a high phagocytic capacity with elevated expression of CD68 (Ziebell et al., 2012). The presence of trains of bipolar/rod-shaped microglia was found in other neurological disorders such as traumatic optic neuropathy, Huntington’s Disease, Parkinson’s Disease and ischemic strokes, and their possible functional roles have been discussed extensively by us and others (Au and Ma, 2017; Holloway et al., 2019; Giordano et al., 2021). This article will therefore review recent advances regarding the roles of bipolar/rod-shaped microglia in AD pathology.

A series of reports highlighted the presence of microglia with highly polarized rod-like morphology in the cerebral cortices, and CA1 and CA2/3 regions of hippocampus during the pathogenesis of AD (Wierzba-Bobrowicz et al., 2002; Bachstetter et al., 2015). These bipolar/rod-shaped microglia are highly proliferative (strong immunoreactivity against a cell proliferation marker PCNA) and colonized in close proximity with senile plaques (Wierzba-Bobrowicz et al., 2002). They also formed end-to-end alignment along with degenerating axons with high immunoreactivity against PHF1 (a marker for NFTs) (Bachstetter et al., 2015). Interestingly, bipolar/rod-shaped microglia in various subcortical regions of AD brains displayed high immunoreactivity against tau protein, suggesting that they might be involved in the internalization of NFTs or degenerating neurons with high loads of hyperphosphorylated tau (Odawara et al., 1995). Similarly, bipolar/rod-shaped microglia were also found in aged chimpanzees with AD-like pathology (Edler et al., 2018), and APP/PS-1 mouse model of AD (Holloway et al., 2020). Interestingly, a recent study demonstrated that the occurrence of bipolar/rod-shaped microglia in cerebral cortex and hippocampus increased with age. The increase in numbers of bipolar/rod-shaped microglia occurred in parietal cortex, but not in hippocampus and temporal cortex in AD-related pathology (Bachstetter et al., 2017). Due to the unique spatial arrangement of bipolar/rod-shaped microglia with damaged axons, it is plausible that bipolar/rod-shaped microglia could exert its neuroprotective action for damaged axons as well as tissue repairing in AD (Giordano et al., 2021).

Ramified and amoeboid microglia can be easily enriched by culturing them onto a fibronectin-coated and laminin-coated surface, respectively (Chamak and Mallat, 1991). However, there is a lack of *in vitro* culture system to enrich bipolar/rod-shaped microglia for molecular or cellular studies in response to cytokines, chemokines, chemoattractant and so forth. We recently established a cost-effective and highly reproducible cell culture system to enrich bipolar/rod-shaped microglia (Tam and Ma, 2014; Tam et al., 2016). Primary microglia purified from postnatal mice cerebral cortices were plated onto a scratched surface pre-coated with poly-D-lysine and laminin, and trains of bipolar/rod-shaped microglia formed stable end-to-end alignment on the laminin-free scratched surface (Tam and Ma, 2014; Tam et al., 2016). These bipolar/rod-shaped microglia were highly proliferative and expressed a decreased levels of M1 (IL-1β, TNF-α, CD32, and CD86) and M2 (TGF-β and IL-10) (Tam and Ma, 2014). In contrast, the microglia colonized in the laminin-rich surface adopted an amoeboid morphology actively digested the laminin surface *via* elevated expression of two laminin-cleaving proteins ADAM9 and CTSS (Tam et al., 2016). Upon LPS stimulation, bipolar/rod-shaped microglia quickly transformed to amoeboid microglia with increased expression of M1 markers (IL-1β and TNF-α) and laminin-cleaving proteins ADAM9 and CTSS (Tam and Ma, 2014; Tam et al., 2016). This cell culture system became a valuable tool to examine the cellular responses of bipolar/rod-shaped microglia toward pathological stimuli, such as Aβ fibrils and NFTs. Interestingly, inhibition of ADAM9 promoted the activity of α-secretase to generate more sAPP-α and thus reduced the production of Aβ (Moss et al., 2011). CTSS is identified as one of the key plaque-induced genes and its up-regulation in microglia might contribute to the formation of Aβ plaques in a mouse model of AD (Chen et al., 2020). Taken together, a switch between amoeboid microglia and bipolar/rod-shaped microglia may contribute to neuroprotection in AD.

## Microglia-Related Risk Factors for Late-Onset Alzheimer’s Disease

Several genetic and environmental factors are known to involve in the pathogenesis of AD (Povova et al., 2012; Xu et al., 2015). Recent large-scale genome-wide association studies reported that several novel genetic variants associated with late-onset AD are related to inflammatory responses, suggesting that microglia are in involved in modulating late-onset AD pathogenesis (Lambert et al., 2013; Karch and Goate, 2015; Sims et al., 2017a). Among the microglia-related genetic factors in late-onset AD, INPP5D, which encodes the inositol polyphosphate-5-phosphatase, is an AD risk gene preferentially expressed in microglia (Lambert et al., 2013). INPP5D is expressed at low level in normal brain; however, the AD-associated INPP5D polymorphism (rs35349669) increased INPP5D gene expression in the brain and whole blood in patients with late-onset AD (Jansen et al., 2017; Tsai et al., 2021). Previous study demonstrated that overexpression of INPP5D inhibited phagocytosis in cultured macrophage cell line (Horan et al., 2007). Interestingly, activated microglia around the Aβ plaques showed increased in INPP5D immunoreactivity in 5xFAD mice (Tsai et al., 2021), suggesting that the reduced phagocytic capacity of microglia might account for the increased Aβ plaques in AD mice. Pharmaceutical blockade of INPP5D and its paralog INPPL1 promoted the internalization of soluble Aβ oligomers and apoptotic neurons in cultured primary microglia (Pedicone et al., 2020).

Notably, a specific rare coding variant (rs72824905, p.P522R) in the gene encoding a microglia-specific phospholipase C gamma 2 (PLCγ2) protein that confers protection against AD (Sims et al., 2017a,b; Magno et al., 2019). Genetic deletion of PLCG2 significantly impaired the microglial phagocytic capacity *via* a TREM2-dependent signaling pathways, suggesting that PLCG2 is required for microglial phagocytosis (Andreone et al., 2020). Overexpression of PLCG2-P522R promoted microglial activation with elevated production of inflammatory cytokines and chemokines (Maguire et al., 2021), and accelerated the clearance of soluble Aβ oligomers in cultured microglia (Takalo et al., 2020; Maguire et al., 2021). Another novel non-synonymous variant ABI3 (rs616338, p.Ser209Phe), increased the risk for the development of late-onset AD (Sims et al., 2017a; Conway et al., 2018; Olive et al., 2020). The ABI3 immunoreactivity was barely detectable in the brains of cognitively normal human; however, ABI3 was preferentially expressed in activated microglia in close proximity to Aβ plaques in AD patients (Satoh et al., 2017; Karahan et al., 2021). Genetic deletion of ABI3 exacerbated Aβ deposition in 5xFAD mice. Since ABI3 is known to regulate actin cytoskeleton organization (Sekino et al., 2015), an important process for microglial migration, deletion of ABI3 might prevent the clustering of active microglia around Aβ plaques and thereby impairing effective Aβ clearance in AD mice (Karahan et al., 2021). More importantly, cultured microglia with ABI3 deletion displayed defective microglial phagocytosis which might also accounted for the increased Aβ deposition in 5xFAD mice (Karahan et al., 2021). It is known that severe niacin deficiency is a cause of dementia, and dietary intake of niacin protected individuals from the development of AD (Morris et al., 2004). Hydroxycarboxylic acid receptor 2 (HCAR2) is a receptor for niacin which is specifically expressed in microglia. A recent study demonstrated that HCAR2 was significantly up-regulated in AD patients and mouse models of AD. Genetic deletion of HCAR2 markedly impaired the uptake of Aβ deposition by microglia, leading to an increase in Aβ deposition and cognitive functional impairment in 5xFAD mice. Interestingly, activation of HCAR2 by using an FDA-approved formulation of niacin (Niaspan) reduced Aβ deposition and neuronal loss, resulting in improved cognitive function in 5xFAD mice (Moutinho et al., 2022), suggesting that HCAR2 activity is required for the effective clearance of Aβ deposition during the early-onset of AD.

## Future Perspective: Therapeutic Interventions Targeting Microglial Activation and Alzheimer’s Disease Pathogenesis

Currently, FDA-approved drugs available for AD patients are mainly cholinesterase inhibitors which partially improve the cognitive function of AD patients (Raina et al., 2008). Several disease-modifying therapies are currently undergoing different phases of clinical trials (Mangialasche et al., 2010); however, most of the anti-amyloid therapies yield little success in phase 3 clinical trials due to the lack of effect on improving cognitive function in AD patients (Huang et al., 2020). For instance, oral administration of CNP520 (a BACE inhibitor) showed worsen cognitive functions in cognitively unimpaired participants with clinical evidence of elevated Aβ loads, leading to early termination of phase 3 clinical trials (Huang et al., 2020). Bapineuzumab is a humanized monoclonal antibody that bind to Aβ monomers, oligomers, and fibrils (Bard et al., 2000). Phase 2 clinical trials showed promising results in reducing Aβ loads and hyperphosphorylated tau proteins in mild-to-moderate AD patients treated with bapineuzumab (Rinne et al., 2010; Blennow et al., 2012). However, no significant improvement in cognitive function was observed in mild-to-moderate AD patients treated with bapineuzumab during a large-scale phase 3 clinical trials (Salloway et al., 2014). In 2019, five on-going clinical trials were conducted to evaluate the efficacy of monoclonal anti-Aβ antibodies (aducanumab, crenezumab, gantenerumab, and solanezumab) (Huang et al., 2020). For aducanumab, the results of phase 3 clinical trials were also disappointing. AD patients treated with aducanumab showed no cognitive improvement (Selkoe, 2019) despite the significant reduction of Aβ plaques in various brain regions (Sevigny et al., 2016). Similarly, solanezumab, gantenerumab and crenezumab failed to prevent cognitive decline and improve cognitive function in mild-to-moderate AD patients during phases 2 and 3 clinical trials (Siemers et al., 2016; Ostrowitzki et al., 2017; Cummings et al., 2018; Honig et al., 2018), suggesting that clearance of Aβ plaques might not be sufficient to reverse neurodegeneration and improve cognitive function in AD patients.

Another therapeutic approach is to target the deposition of NFTs that made up of hyperphosphorylated tau (Congdon and Sigurdsson, 2018). Several tau aggregation inhibitors have been developed and currently undergoing clinical trials at different phases. Leuco-methylthioninium (LMTX) was a second-generation tau aggregation inhibitor that are undergoing phase 3 clinical trials. Interestingly, one phase 3 clinical trial on low-dose LMTX monotherapy showed some cognitive improvement in mild-to-moderate AD patients. Currently, a third phase 3 clinical trial is still on-going with the results expected to complete in early 2023 (Marasco, 2020). Two tau aggregation inhibitors (AADvac1 and zagotenemab) are currently undergoing phase 2 clinical trial; however, the clinical outcomes are yet to determined. Treating transgenic rats expressing truncated tau proteins with AADvac1 effectively reduced the amount of NFTs deposition in the brain (Novak et al., 2019). Transgenic mice expressing mutant tau proteins treated with MC-1 (derivatives of zagotenemab) greatly reduced the amount of hyperphosphorylated tau and NFTs in the forebrain (Chai et al., 2011). A recent study highlighted the importance of NFTs formation in reducing gray matter volume and inducing cognitive function impairment in AD patients (Bejanin et al., 2017). The severity of cognitive impairment in AD patients is well-correlated with the density of NFT aggregates (Nelson et al., 2012; Bejanin et al., 2017). However, the density of Aβ plaques might not correlate well with the severity of dementia (Nelson et al., 2012). Therefore, NFTs formation-related processes that are potential targets for therapeutic intervention would have better clinical outcomes than do therapies targeting Aβ production (Congdon and Sigurdsson, 2018).

Microglial transcriptomic analysis demonstrate that microglial activation often precedes the deposition of Aβ plaques and NFTs in AD patients and mouse models of AD (Keren-Shaul et al., 2017; Mathys et al., 2017; Friedman et al., 2018; Chen et al., 2020). By modulating the phenotypic states of microglia at the early stage of AD might be considered as an effective therapeutic approach to slow down or even stop disease progression. In homeostatic conditions, microglia are heterogeneous and functionally diverse with high expression of homeostatic microglia signature genes such as *Tmem119* and *P2ry12* (Masuda et al., 2019). Although the functions of homeostatic microglia signature genes remain largely unknown, dramatic decline in homeostatic markers indicates a loss of homeostatic microglial function in the progression of AD (Keren-Shaul et al., 2017; Mathys et al., 2017; Masuda et al., 2019), More importantly, the transition from homeostatic microglia to DAM involved up-regulation of *Trem2* and *Apoe* (Keren-Shaul et al., 2017; Masuda et al., 2019; Chen et al., 2020; Olah et al., 2020; Srinivasan et al., 2020), which are considered as the major risk factors for AD (Roses, 1996; Cheng-Hathaway et al., 2018; Sudom et al., 2018). The precise role of Trem2 in AD pathogenesis still remains to be highly controversial despite several studies showing that deletion of *Trem2* restored homeostatic microglia signature gene expression (Keren-Shaul et al., 2017; Krasemann et al., 2017) and reduced Aβ deposition in APP/PS1 mice (Jay et al., 2015; Krasemann et al., 2017). In contrast, another studies reported that deletion of *Trem2* or *Trem2* haploinsufficiency augmented Aβ deposition in both APP/PS1 and 5xFAD mice (Wang et al., 2015b; Jay et al., 2017). However, it is now clear that ApoE has a detrimental role during AD pathogenesis. In fact, the expression of ApoE was markedly increased in microglia adjacent to Aβ plaques (Krasemann et al., 2017; Chen et al., 2020). ApoE is known to induce transcription of APP and increase Aβ synthesis in cultured neurons (Huang et al., 2017), and facilitate the aggregation of Aβ (Kim et al., 2009). Genetic ablation of ApoE restored homeostatic microglia signature gene expression, and protected the neurons from apoptosis 7 days after facial nerve axotomy (Krasemann et al., 2017). Pharmaceutical blockade of ApoE by neutralizing antibodies against ApoE promoted microglial phagocytosis of Aβ plaques and reduced the production of pro-inflammatory cytokines (i.e., IFN-γ and IL-1α) in APP/PS-1 mice (Kim et al., 2018). Similarly, treating young APP/PS-1 mice with antisense oligonucleotides (ASOs) that specifically reduced ApoE expression, Aβ pathology was significantly reduced at 16 weeks (Huynh et al., 2017). To this end, these studies highlight the fact that ApoE might serve as an attractive therapeutic target for the prevention of AD.

Several therapeutic interventions that aim to reduce neuroinflammation in the early stage of AD are currently at various phases of clinical trials for AD (Huang et al., 2020). Pioglitazone, agonist of the nuclear receptor peroxisome proliferator-activated receptor γ (PPAR-γ), which is known to reduce the gene expression of pro-inflammatory cytokines, and has been shown to be effective to slow down Aβ deposition and improve cognitive function in MCI patients (Sato et al., 2011). Azeliragon, a small-molecule inhibitor of receptor for advanced glycation end-products (RAGE), is able to reduce Aβ deposition, ameliorate the production of IL-1 and TNF-α, and improve spatial memory function in a mouse model of AD (Burstein et al., 2018). Phase 2 clinical trials demonstrated that azeliragon delayed cognitive decline in mild-to-moderate AD patients (Burstein et al., 2018). Taken together, modulation of the phenotypic states of microglia exhibit significant therapeutic potential to slow down AD progression given that further investigation is required to determine optimal timing of intervention. With the exponential growth in the availability of microglial transcriptomic data, researchers can take this opportunity to perform in-depth *in silico* drug screening to identify bioactive small molecules that can restore the homeostatic microglia and inhibit ApoE-mediated signaling pathways for the effective treatment of AD (Lamb et al., 2006; Vargas et al., 2018).

## Author Contributions

YJX, NPBA, and CHEM discussed and formulated the focus of the review. YJX and NPBA conducted the literature search and drafted the manuscript under the supervision of CHEM. CHEM evaluated and revised the manuscript for final submission. All authors have read and approved the final version of manuscript for submission. Schematic illustrations are created with BioRender.com.

## Conflict of Interest

The authors declare that the research was conducted in the absence of any commercial or financial relationships that could be construed as a potential conflict of interest.

## Publisher’s Note

All claims expressed in this article are solely those of the authors and do not necessarily represent those of their affiliated organizations, or those of the publisher, the editors and the reviewers. Any product that may be evaluated in this article, or claim that may be made by its manufacturer, is not guaranteed or endorsed by the publisher.
